# Gender biased neuroprotective effect of Transferrin Receptor 2 deletion in multiple models of Parkinson’s disease

**DOI:** 10.1038/s41418-020-00698-4

**Published:** 2020-12-16

**Authors:** Chiara Milanese, Sylvia Gabriels, Sander Barnhoorn, Silvia Cerri, Ayse Ulusoy, S. V. Gornati, Daniel F. Wallace, Fabio Blandini, Donato A. Di Monte, V. Nathan Subramaniam, Pier G. Mastroberardino

**Affiliations:** 1 Department of Molecular Genetics, Rotterdam, the Netherlands; 2 IFOM-The FIRC Institute of Molecular Oncology, Milan, Italy; 3 IRCCS Mondino Foundation, 27100 Pavia, Italy; 4 German Centre for Neurodegenerative Diseases (DZNE), 53175 Bonn, Germany; 5 Department of Neuroscience Erasmus MC, Rotterdam, the Netherlands; 6 School of Biomedical Sciences, Institute of Health and Biomedical Innovation, Queensland University of Technology (QUT), Brisbane, QLD Australia; 7 Department of Brain and Behavioral Sciences, University of Pavia, Pavia, Italy; 8 Department of Life, Health, and Environmental Sciences, University of L’Aquila, L’Aquila, Italy

**Keywords:** Metals, Ageing, Neurological disorders

## Abstract

Alterations in the metabolism of iron and its accumulation in the *substantia nigra pars compacta* accompany the pathogenesis of Parkinson’s disease (PD). Changes in iron homeostasis also occur during aging, which constitutes a PD major risk factor. As such, mitigation of iron overload via chelation strategies has been considered a plausible disease modifying approach. Iron chelation, however, is imperfect because of general undesired side effects and lack of specificity; more effective approaches would rely on targeting distinctive pathways responsible for iron overload in brain regions relevant to PD and, in particular, the substantia nigra. We have previously demonstrated that the Transferrin/Transferrin Receptor 2 (TfR2) iron import mechanism functions in nigral dopaminergic neurons, is perturbed in PD models and patients, and therefore constitutes a potential therapeutic target to halt iron accumulation. To validate this hypothesis, we generated mice with targeted deletion of TfR2 in dopaminergic neurons. In these animals, we modeled PD with multiple approaches, based either on neurotoxin exposure or alpha-synuclein proteotoxic mechanisms. We found that TfR2 deletion can provide neuroprotection against dopaminergic degeneration, and against PD- and aging-related iron overload. The effects, however, were significantly more pronounced in females rather than in males. Our data indicate that the TfR2 iron import pathway represents an amenable strategy to hamper PD progression. Data also suggest, however, that therapeutic strategies targeting TfR2 should consider a potential sexual dimorphism in neuroprotective response.

## Introduction

Parkinson’s disease (PD) is a neurodegenerative disorder primarily affecting dopaminergic (DA) neurons in the nigrostriatal circuits of the basal ganglia. The disorder is mainly idiopathic and less than 10% of cases are attributable to monogenic causes. PD etiopathology is intricate and involves complex gene–environment interactions perturbing cellular functions at multiple levels [1]; the main disease risk factor is aging [2, 3]. Mitochondrial dysfunction, alpha-synuclein aggregation and consequent proteotoxic stress, and noxious oxidant stress have been identified as central pathogenic mechanisms [4]. At present, however, PD remains intractable and therefore identification of specific pathways that can be targeted to modify disease progression remains of primary importance.

Perturbation of iron metabolism and consequent deposition of iron in the *substantia nigra pars compacta* (SNpc) and in its DA neurons occur in both idiopathic and genetic PD [5–9]. These alterations may have profound neuropathological relevance; iron is essential for the function of mitochondria [10], it promotes aggregation of alpha-synuclein [11], and favors production of oxidant species [12].

Increasing evidence indicates that, rather than being a mere epiphenomenon, iron dysregulation actively contributes to PD pathogenesis. Chelation experiments to reduce iron bioavailability, for instance, successfully oppose neuronal damage in animal models [13, 14]. Clinical evidence further supports this relationship and recent phase II clinical trials demonstrated that administration of the iron chelator deferiprone effectively lowered motor decline in patients [13, 15]. Altogether this evidence indicates that restoration of iron homeostasis may represent an effective therapy for PD. Iron chelation, however, is an imperfect strategy to restore iron homeostasis, at least in this particular disorder. Besides its general adverse side effects [16], iron chelation has been conceived to treat diseases with systemic iron overload, which does not occur in PD. Because body and brain total iron levels are unaltered in PD, PD iron changes rather result from a selective accumulation of the metal in the SN [17]. Thus, while systemic chelation may result in depletion of bioavailable iron with severe consequences for the organism, iron overload in PD may be better treated by targeting those mechanisms specifically responsible for metal accumulation in the SNpc.

We have previously used a pesticide-induced PD model to investigate the effects of PD-related environmental agents on iron metabolism in rats and nonhuman primates [18]. We found that toxin-induced neurodegeneration is associated with progressive increase in the levels and oxidation of transferrin (Tf)—the principal iron transporter in the body—within DA neurons. Such increase is paralleled by iron accumulation. Intracellular levels of Tf reflect the trafficking mediated by its receptors and, thus far, two receptor isoforms have been identified. Transferrin receptor 1 (TfR1) has an iron responsive element (IRE) in its 3′UTR and therefore, is directly regulated by intracellular iron levels; on the other hand, the Transferrin Receptor 2 (TfR2) does not possess an IRE and is regulated by means other than iron, possibly involving the hormone hepcidin [19, 20]. Analyses of both tissue from PD animal models and autoptic tissue from patients revealed that TfR2, rather than TfR1, is highly expressed in DA neurons [18]. Importantly, we also demonstrated that, within DA neurons, the Tf/TfR2 system delivers iron to mitochondria via endosome-mitochondria interactions, possibly via a kiss-and-run mechanism as described in other cell types [21–23]. Based on these findings, we concluded that the Tf/TfR2 axis is a major mechanism for iron transport into DA neurons and hypothesized that its perturbation may enhance vulnerability to neurodegenerative processes and therefore act as a PD risk factor. Support in favor of this hypothesis came from the results of an epidemiological study that analyzed three different PD cohorts to explore association between variants in genes involved in iron metabolism and PD [24]. Tf and TfR2 were the only two genes showing, respectively, significant and suggestive significant—as defined in [25]—protective association with PD; importantly, in one of the cohorts, TfR2 reached significance. This genetic study substantiates our previous laboratory discoveries in animal models and PD brain autopsy tissues, and further strengthen the hypothesis that the Tf/TfR2 axis may participate in PD pathogenesis.

To further test the relevance of the Tf/TfR2 iron import mechanism to PD pathogenesis, we modeled the disease in control mice and mutant animals with targeted TfR2 deletion in neurons expressing tyrosine hydroxylase (TH)—including SNpc DA neurons. In these mice, degeneration of nigral neurons was achieved after exposure to the neurotoxicant MPTP, overexpression of α-synuclein (a protein involved in PD pathogenesis) or intraparenchymal injections of pre-formed alpha-synuclein fibrils (PFF). We treated male and female animals as different experimental groups because clinical evidence and large data meta-analysis demonstrate sexual dimorphism in PD and gender differences are also seen iron metabolism [26–28]. Here, we demonstrate that TfR2 ablation provides neuroprotection in animal models of PD and that this effect is gender-dependent and significantly more pronounced in female as compared to male mice.

## Results

### Generation of TfR2 conditional mutants and PD modeling

**Fig. 1:**
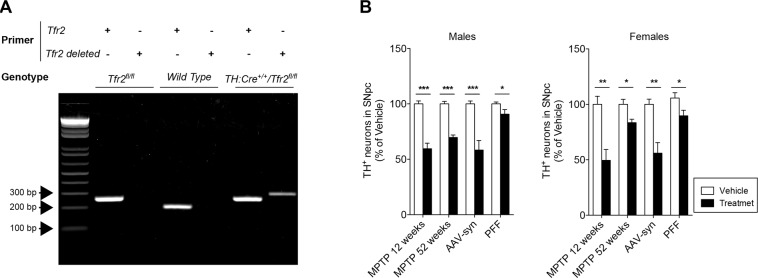
Targeted *TfR2* deletion in TH expressing cells and PD modeling in mice. **A** PCR to confirm *TfR2* deletion performed on genomic DNA extracted from 30-μm-thick PFA-fixed sections of the olfactory bulbs, which abound in TH-neurons. DNA from two consecutive slices was used in each PCR reaction. Genotyping was performed using two different sets of primers, one of which (TfR2) amplifies the WT or floxed allele, while the other (TfR2 deleted) amplifies the deleted allele. Selective recombination of *TfR2* selectively occurs only in Cre-expressing floxed mice (*Tfr2*^fl/fl^TH:Cre^+/−^, last lane) and not in mice lacking Cre expression (lanes 2 and 4). **B** Validation of the PD-related treatments used in the study, which successfully elicited dopaminergic degeneration, as indicated by unbiased stereological counts. Graphs represent mean + SEM, two-tailed Student’s *t* test; *p < 0.05, **p < 0.005, ***p < 0.001.

Mutant mice with targeted deletion of TfR2 were generated using the recombinase Cre:loxP system and crossing TfR2-floxed mice [29] with a transgenic mice expressing the recombinase under the TH promoter [30]. In the derived strain (*TfR2*^fl/fl^:TH-Cre^+/−^), *TfR2* is deleted in catecholaminergic neurons, including dopamine neurons of the SNpc. Because Cre-driven recombination may be variable even among littermates [31], gene deletion was confirmed in every single mouse used in the study by PCR on genomic DNA [29] extracted from the olfactory bulb, which is rich in DA neurons (Fig. 1A and Supplementary Fig. 1A). Moreover, breeding pairs were set up so that the *Cre*^*+*/*−*^ transgene was always transmitted by the mother because recombination activity may vary depending upon the gender of the supplying parent [31].

The different PD-related treatments involved in the study–i.e., sub-acute MPTP injection, rAAV-mediated human alpha-synuclein overexpression (rAAV), and infusion of PFF—successfully induced DA degeneration, as indicated by unbiased stereological counts (Fig. 1B). Because aging is a main risk factor for PD, PD-like neurodegeneration was also induced by MPTP administration to 52-week-old animals. Initial experiments revealed that the MPTP paradigm used in younger animals (30 mg/kg) induced very high mortality (<80%) in older mice. In the latter, we therefore used a lower MPTP dose (17.5 mg/kg) that elicited significant DA cell degeneration with no or minimal mortality.

### Targeted *TfR2* deletion in TH^+^ cells alleviates neuropathology in the MPTP model

**Fig. 2:**
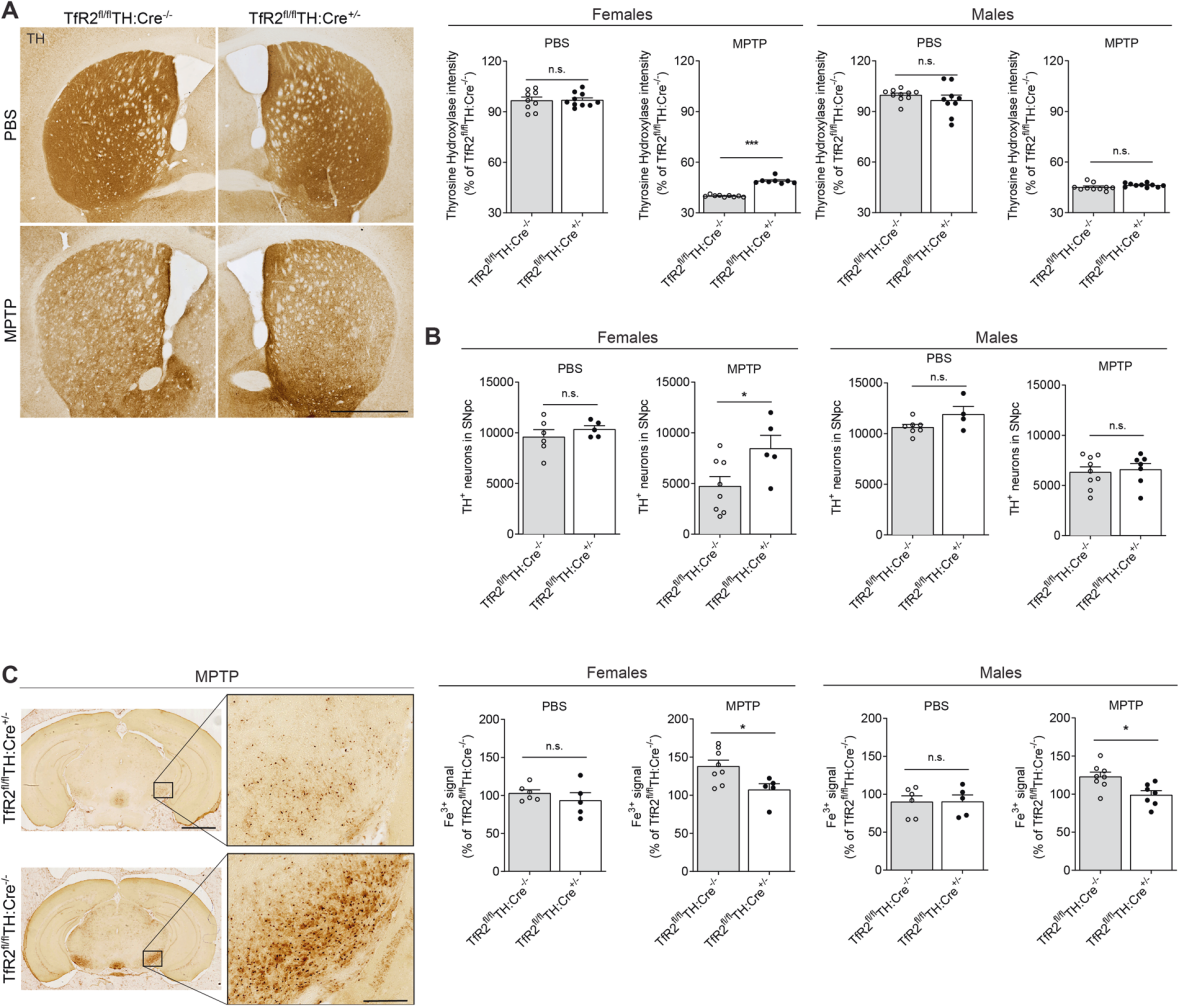
*TfR2* deletion is protective in 12-week-old MPTP-treated mice. **A** TH immunoreactivity in striata of MPTP or vehicle-treated animals. *TfR2* deletion mitigates striatal denervation in brains of female mice. **B** Unbiased stereological counts of TH immunopositive neurons in the substantia nigra confirm that *TfR2* targeted deletion is protective in 12-week-old females, but not in males. **C** Perls’ staining for Fe^3+^ reveals reduced iron accumulation in the midbrain after MPTP treatment in animals with TfR2 targeted deletion (12-week-old mice). Data in experimental groups are expressed as percentage of the respective *TfR2*^fl/fl^TH:Cre^−/−^ vehicle-treated control group. Two-tailed Student’s *t* test; *p < 0.05, **p < 0.005, ***p < 0.001. Calibration bars: 1 mm in (**A**), 2 mm (left) and 100 μm (right) in (**C**).

In 12-week-old female mice, *TfR2* deletion caused significant neuroprotection, as evidenced by increased immunoreactivity of striatal TH (Fig. 2A) and higher counts of nigral DA cell bodies in *TfR2*^fl/fl^:TH-Cre^+/−^ mice (Fig. 2B). These effects did not occur, however, in male mice, in which no evidence of neuroprotection was observed at the levels of both striatal terminals and nigral cell bodies. Perls staining to evaluate Fe^3+^ levels revealed that *TfR2* deletion led to a significant reduction of ferric iron in both MPTP-treated males and females (Fig. 2C).

**Fig. 3:**
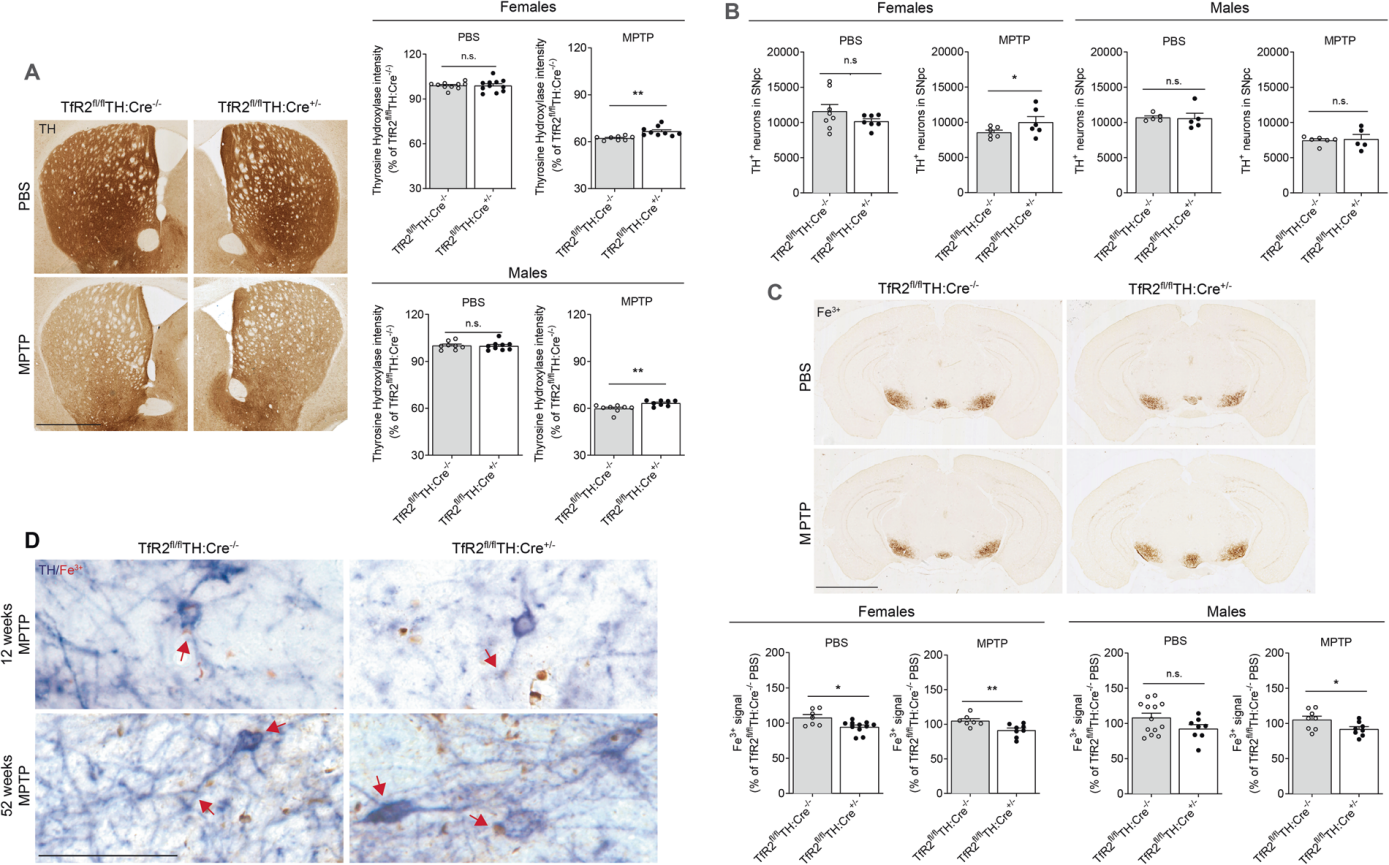
*TfR2* deletion is protective in 52-week-old MPTP-treated mice. **A** Striatal TH immunoreactivity in 52-week-old mice. *TfR2* deletion provides moderate, yet significant protection from MPTP treatment**. B** Unbiased stereological counts of TH immunopositive neurons in the SNpc confirm that *TfR2* deletion is protective in females, but not in males. **C**. Perls’ staining for Fe^3+^ reveals significant decrease in iron accumulation in the substantia nigra in after MPTP treatment in animals with TfR2 targeted deletion. In 52-week-old mice, *TfR2* deletion also reduces accumulation of iron deposits in untreated females. **D** Double histological staining to reveal Fe^3+^ in TH immunopositive neurons in the SNpc, in 12- and 52-week-old mice. Iron deposits partially co-localize with dopaminergic neurons (arrows). Iron levels are remarkably more evident in 52-week than in 12-week-old-treated animals. All data are expressed as percentage of the *TfR2*^fl/fl^TH:Cre^−/−^ vehicle-treated control group. Two-tailed Student’s *t* test; *p < 0.05, **p < 0.005, ***p < 0.001. Calibration bars: 1 mm in (**A**), 2 mm in (**C**) and 50 μm in (**D**).

Findings in 52-week-old female mice were similar to those in younger animals; following MPTP exposure, TfR2 deletion ameliorated neuropathology, as indicated by enhanced striatal innervation (Fig. 3A), and a higher number of DA cell bodies in the SNpc of *TfR2*^fl/fl^:TH-Cre^+/−^ mice (Fig. 3B). In MPTP-treated males, TfR2 ablation slightly improved striatal innervation, but had no effect on DA cell body loss.

In agreement with earlier studies [32], older control mice—vehicle-treated—displayed higher basal ferric iron levels in the ventral mesencephalon when compared to younger animals (Fig. 3C). Similar to the results in younger mice, reduced iron accumulation was observed in *TfR2*^fl/fl^:TH-Cre^+/−^ in both MPTP-treated females and males. Double staining – i.e. Perls’ staining combined with TH immunocytochemistry - confirmed increased iron levels within DA neurons of the SNpc (Fig. 3D); this effect was remarkably more evident in 52- week than in 12-week-old animals.

In conclusion, targeted deletion of *TfR2* reduced toxin- and age-induced ferric iron accumulation in the ventral mesencephalon. It also conferred neuroprotection against MPTP-induced nigrostriatal damage, but did so primarily in young and old female mice.

### Effects of targeted *TfR2* deletion in TH^+^ cells in synucleinopathy models of PD

We next evaluated the protective potential of TfR2 deletion in two synucleinopathy models, namely in (1) mice in which human alpha-synuclein overexpression was induced by a stereotaxic injection of recombinant adeno-associated (rAAV) viral vectors in the substantia nigra [33], and (2) mice in which alpha-synuclein PFF were stereotaxically injected in the striatum [34].

**Fig. 4:**
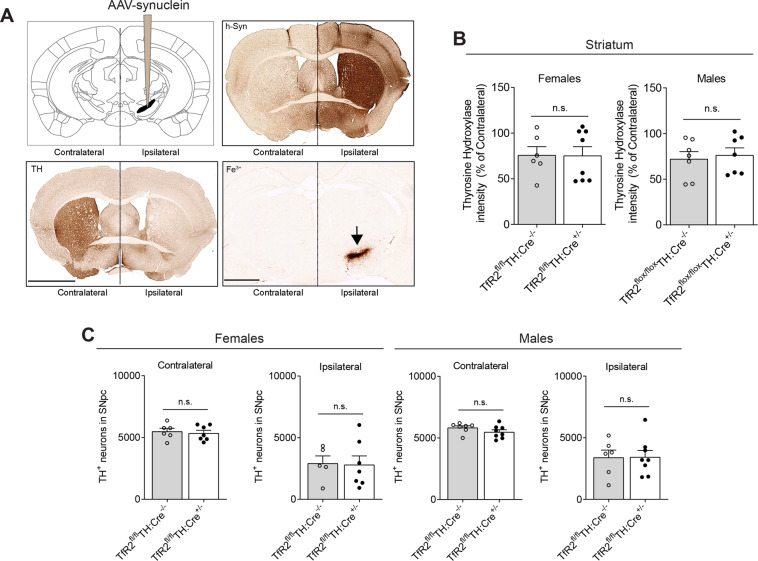
Effects of *TfR2*-targeted deletion in an AAV-h-alpha-synuclein overexpressing model of PD. **A** Schematic of intracranial injection of AAV-h-synuclein in the right SNpc hemisphere (ipsilateral). Transduction with rAAV coding for human alpha-synuclein (h-syn) results in its striatal increase in the ipsilateral hemisphere, as showed in the top left panel that is paralleled by decreased TH levels (bottom left panel) and remarkable Fe^3+^ deposits accumulation at the site of injection in the SNpc, indicative of ongoing pathology. **B**, **C** *TfR2* deletion does not provide any protection against AAV-h-synuclein overexpression, as indicated by striatal dopaminergic innervation expressed as percentage of the contralateral side (**B**) and unbiased stereological counts of dopaminergic neurons in the SNpc. Graphs represent scatter plots with bars. Two-tailed *t* test. Calibration bars: 1 mm (top) and 2 mm (bottom) in (**A**).

As expected, rAAV-transduced mice displayed a conspicuous accumulation of striatal human alpha-synuclein (i.e., exogenous alpha-synuclein coded by rAAVs) in the ipsilateral hemisphere that was paralleled by a considerable reduction of DA innervation (Fig. 4A). Measurements of striatal TH levels on the side of the brain ipsilateral to the rAAV injection showed no significant differences between mice with and without *TfR2* deletion (Fig. 4B). Similarly, stereological counts of ipsilateral nigral neurons revealed a loss of cells that was comparable between control and *TfR2*^fl/fl^:TH-Cre^+/−^ animals (Fig. 4C). Of note, ipsilateral TH and cell count values were quite variable, probably reflecting the variability of human alpha-synuclein expression among rAAV-injected animals (Supplementary Fig. 2).

Assessment of iron levels in this rAAV model was relatively inconsistent, mostly because the Fe^3+^ Perls’ staining often marked the site and followed the track of stereotaxic injection, even at 6 months after surgery (Supplementary Fig. 3, arrowheads). In a few animals, iron staining revealed a pattern of nigral accumulation (Fig. 4A, Supplementary Fig. 3, asterisks) whereas, in the other mice, iron deposition was less evident in the ventral midbrain, and the signal was primarily present along the needle track (arrow).

**Fig. 5:**
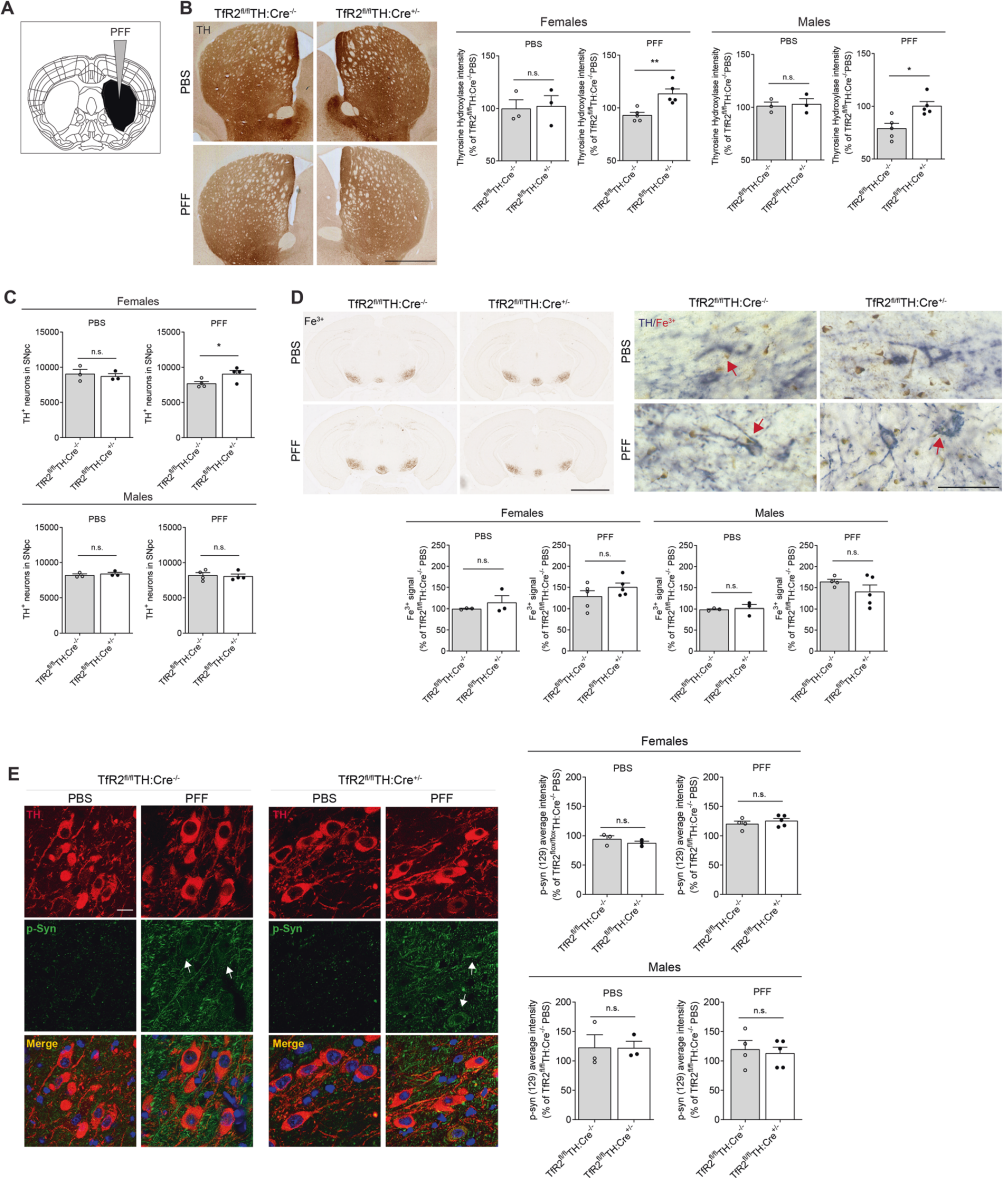
*TfR2*-targeted deletion is partially protective against alpha-synuclein pre-formed fibrils (PFF) intracranial seeding. **A** Schematic of the stereotactic injection procedure during which PFF have been injected in the right striatum. **B** *TfR2* deletion provides protection against striatal denervation induced by PFF, as indicated by TH immunohistochemistry. **C** Unbiased stereological counts of dopaminergic neurons in the SNpc indicate that *TfR2*-targeted deletion is protective against PFF-induced degeneration in females, but not in males. **D** Perls’ staining for Fe^3+^ did not reveal any effect of *TfR2* deletion on iron accumulation in the SNpc induced by PFF (left panel). Immunodetection of TH-positive neurons combined with Perls’ staining reveals iron deposits also in SNpc dopamine neurons (right panel). **E** Increased levels of ser129 p-α-synuclein in the substantia nigra (arrows) confirms α-synuclein stress in PFF-injected animals that is not ameliorated by *TfR2* deletion (green signal). Graphs represent scatter plots with bars. Two-tailed *t* test; *p < 0.05, **p < 0.005, ***p < 0.001. Calibration bars: 1 mm in (**B**), 2 mm (left panel) and 50 μm (right panel) in (**D**), and 50 μm in (**E**).

The final set of experiments was aimed at evaluating the effects of *TfR2* deletion in mice in which nigral cell degeneration was triggered by a stereotaxical injection of alpha-synuclein PFF in the striatum (Fig. 5A). As compared to the rAAV model, the paradigm of striatal PFF infusion is characterized by a nigrostriatal injury that is more moderate and evolves in a relatively more progressive fashion [34]. These features were taken into consideration when we decided to perform our analyses at 4 months after the PFF injection. Data showed that *TfR2* deletion was associated with an increase in striatal TH immunoreactivity in both female and male mice and with a significant protective effect against nigral DA cell loss only in female animals (Fig. 5B, C). Perls’ staining combined with immunodetection of TH-positive neurons showed increased iron levels within SNpc dopamine neurons as a result of PFF administration. No differences in total iron levels were detected, however, between *TfR2*^fl/fl^:TH-Cre^+/−^ and *TfR2*^fl/fl^:TH-Cre^−/−^ PFF-treated animals (Fig. 5D). Ser129 phospho-synuclein was also assessed in SNpc DA neurons as a marker of ongoing alpha-synuclein aggregate pathology. Immunoreactivity for phospho-synuclein was markedly increased in PFF-injected mice, but the extent of this increase was not significantly affected by *TfR2* deletion (Fig. 5F).

In summary, these results indicate that targeted *TfR2* deletion had neuroprotective effects mostly in female mice in a synucleinopathy model triggered by PFF injections. This neuroprotection occurred in the absence of overt reduction of iron accumulation and despite an overload of phosphorylated alpha-synuclein.

## Discussion

Iron is critical for multiple processes implicated in PD pathobiology such as mitochondrial function and oxidant stress. In addition, iron is involved in DNA damage and repair—being utilized as a cofactor for repair enzymes [35] as well as catalyzing obnoxious lesions in the double helix—which also participate in PD pathogenesis and are mechanistically associated with aging, i.e., PD principal risk factor [36–39]. Convergent evidence suggests that intervention to prevent iron overload in the SNpc may be a promising disease modifying approach [40]. Regional increase of iron in the SNpc of PD patients has been robustly documented in independent studies [7, 41–44] and further substantiated by a recent meta-analysis [45]. Consistently, recent clinical trials demonstrated the efficacy of the iron chelator deferiprone [13, 15]. Because chelation strategies are imperfect [16], identification of a specific pharmacological target responsible for iron accumulation could lay the foundation for more precise intervention.

It is recognized that biomedical research, and neuroscience in particular, suffer from a gender bias that should be urgently corrected to achieve more comprehensive understanding of disease mechanisms [46–48]. In this study, we therefore treated male and female animals as separated experimental groups. Our most salient data demonstrate that deletion of TfR2 in catecholaminergic cells—and thus also in the dopamine neurons of the SNpc—mitigates neuropathology in oxidative stress and proteotoxic models of PD and reduces the age-related iron increase in the SNpc. Intriguingly, these effects display gender dimorphism and are detectable only in females.

The precise mechanisms underlying the differences in male versus females mice remain to be addressed; the phenomenon, however, is perfectly consistent with gender dimorphism in both iron metabolism [28, 32, 49] as well as in PD prevalence and, to some extent, clinical presentation [50]. A plausible hypothesis is that TfR2 deletion synergizes with female sex hormones, which are known to mitigate iron-mediated as well as general cellular oxidant stress, also when induced by PD-related toxins such as paraquat [27, 51–53]. This hypothesis can be tested in future studies via administration of estrogens or castration in males and in ovariectomized females. Indeed, multiple studies demonstrated a neuroprotective effect of estrogen in the MPTP and 6-hydroxydopamie PD models. Here, the underlying mechanisms include modulation of the renin–angiotensin system and of NADPH oxidase activity, possibly culminating in different activation patterns of microglia and astrocytes to favor protective over detrimental subtypes [54–59].

The biological reliance of DA neurons on TfR2 and its consequent role in PD may be related to the distinctive physiological properties of these cells. Unlike TfR1, TfR2 expression is not reduced upon iron overload by post-transcriptional mRNA regulation [60] and therefore may maintain sustained import activity also at high intracellular iron concentrations. Dopamine neurons may have particular high iron demand as evidenced, for instance, by the role of iron as a cofactor in the highly abundant TH enzyme, which catalyzes the rate limiting step in dopamine biosynthesis, as well as by the presence of neuromelanin, which functions as an additional, peculiar iron buffering system [61]. Under these circumstances, an import mechanism independent of iron concentration may guarantee effective uptake also when iron levels are relatively elevated. Dopamine neurons, however, also display high basal oxidation, which is intrinsically associated with their physiological properties [62, 63]. We speculate that this counterintuitive and potentially harmful biological combination—i.e. elevated iron, an iron-insensitive import mechanism, and high oxidation—is an example of antagonistic pleiotropy. These physiological properties may in fact be instrumental for providing tight regulation of redox signaling—which is essential for SNpc DA neurons—and thus of the associated behavior. Overall, these features may offer an advantage during reproductive stages of life while becoming detrimental during aging [64]. If not carefully regulated, for instance because of aging-related deterioration, this distinctive physiological layout may easily lead to harmful consequences, which may be in turn be mitigated by specific reduction of TfR2-mediated iron uptake.

Our results are also relevant in light of emerging evidence indicating that ferroptosis [65]—an iron-dependent programmed cell death pathway—may participate in PD pathogenesis. Ferroptosis, in fact, is associated with cardinal PD features such as redox imbalance and mitochondrial dysfunction [66] and requires Tf-mediated iron import [67]. Hampering Tf import to prevent iron overload in DA neurons may therefore provide protection against this type of cell death mechanism.

Neuroprotection in both young and old MPTP-treated mice is in agreement with the notion that iron dysregulation and accumulation may promote oxidative damage via Fenton chemistry. Protection could occur through redox-related mechanisms also in the PFF model, consistent with documented alpha-synuclein ability to promote oxidation [68]. In fact, the lack of an effect on phospho-synuclein levels in TfR2 mutants suggests that TfR2 deletion primarily affects mechanisms other than alpha-synuclein stress. However, additional pathogenic mechanisms specifically related to alpha-synuclein proteotoxic stress may explain neuroprotection in the PFF model despite lack of differences in iron deposition. For instance, recent evidence revealed an interaction between alpha-synuclein oligomerization and stress, and genes involved in the endocytic pathway [69]. Alterations in endocytosis in the PFF model—for instance in an attempt to remove toxic oligomers—may therefore be paralleled by undesired increased uptake of iron. Further studies will be necessary to elucidate the mechanisms underlying differences in iron accumulation in models of PD-like neurodegeneration based on oxidant or proteotoxic stress. The negative result observed in the rAAV mouse model may stem from the high variability observed in this study, which is consistent with previous observations using this model [70].

Neuroprotective effects mediated by TfR2 deletion were more evident at the striatal level rather than in the SNpc, with improvement in striatal innervation, but no corresponding effect on DA cell body loss. These differential observations in the striatum and SNpc may be consistent with the concept that, in PD and PD models, neuritic pathology precedes frank cell death [71–73]. In addition, the neuroprotective effect of TfR2 deletion against MPTP is more pronounced in younger as compared to older female mice; this evidence is consistent with other reports indicating that, in the MPTP model, neuroprotective molecules that may indirectly reduce oxidant stress, for instance acting on dopamine metabolism, are more effective in younger animals [74].

While the protective effect of TfR2 deletion is moderate, several elements suggest that its magnitude may improve by repeating the study in different organisms. For instance, the biosynthetic pathway of ascorbate, which is also involved in the brain redox regulation, is intact in mice, while it is lost in primates and humans [75]. Performing the experiments in organisms lacking the capacity of ascorbate synthesis might potentiate the effects of TfR2 deletion. In addition, iron metabolism differs among mice strains, most likely because of genetic factors and, for instance, non-heme iron levels are reduced in some tissues of C57BL/6 mice when compared to other strains [76]. Repeating the study on a different strain might amplify the effect of TfR2 deletion, which could also extend the neuroprotective effect to males, therefore circumventing the gender issue.

Perls’ staining combined with specific immunodetection of different cell types to study iron accumulation with cellular resolution showed iron accumulation in DA neurons, but did not detect differences between wild type and *TfR2*^fl/fl^:TH-Cre^+/−^ mutant mice. These results may point to insufficient sensitivity of this method. Iron deposition outside TH^+^ cells may be explained, at least in part, by previous evidence from our laboratory indicating that DA degeneration—which is associated with increased intracellular oxidation, possibly caused by iron overload—is paralleled by a loss in TH phenotype [77]. Collectively these elements suggest that, at the observed time points, cells with detectable ferric iron may have already lost their neurochemical phenotype. Alternative methods with higher sensitivity will be required to conclusively address these issues.

Finally, in male animals reduction in iron levels was not paralleled by neuroprotection. While this evidence could be again attributed to limited sensitivity of the Perls’ method, it could also suggest an iron-independent role for TfR2 in PD progression. TfR2 is relatively poorly characterized and, at present and to our best knowledge, potential roles for this protein outside the iron trafficking domain are unknown. Further, in depth studies will be therefore necessary to fully unravel the mechanisms connecting TfR2, iron levels, and PR progression.

In conclusion, our study identifies a tractable target to mitigate neurotoxic iron overload in DA neurons during PD pathogenesis and therefore lays the foundation for new disease modifying therapies.

## Materials and methods

### Chemicals

All reagents were purchased from Sigma-Aldrich unless otherwise specified.

### Animal care

Animals were kept on a regular diet and housed at the Animal Resource Center (Erasmus University Medical Center), which operates in compliance with the “Animal Welfare Act” of the Dutch government, following the “Guide for the Care and Use of Laboratory Animals” as its standard.

Transgenic mice expressing recombinase Cre under the TH promoter [30] were purchased from The Jackson Laboratory, crossed with TfR2fl/fl mice [29] and kept on the C57Bl/6J background. Males and females have been considered as separate experimental groups because in light of gender dimorphism in iron metabolism [28, 32, 49], PD prevalence [50], and different sensitivity to the MPTP drug [78]. Initially, the size of the experimental groups was determined by power analysis; however, in compliance with the reduction criterion in animal experimentation and to limit the use of mice, the number of animals in the experiment group was maintained to the minimal size required to achieve significance in the primary outcome measures, i.e., striatal innervation and stereological counts.

### Genomic PCR to confirm TfR2 deletion

To confirm the occurrence of the targeted TfR2 deletion, genomic DNA analysis was performed on each TfR2^fl/fl^TH:Cre^+/−^ double mutant and TfR2^fl/fl^TH:Cre^−/−^ mouse used in the study. Briefly, DNA was extracted from three consecutive 30-µm-thick paraformaldeyde (PFA 4%) fixed sections derived from the olfactory bulb area [79]. Sections were incubated in 100 mM EDTA, 50 mM Tris-HCl, 100 mM NaCl, 1% SDS and 0.15 mg/mL proteinase K for 72 h at 65 °C in agitation and protected from light. After 5 min of centrifugation at 4 °C and 10,000 rpm, the liquid phase was purified with an equal volume of phenol–chloroform–isoamyl alcohol (25:24:1) and centrifuged for 10 min at 10,000 rpm. The liquid phase was then washed with chloroform–isoamyl alcohol (24:1) and then precipitated with 1/10 3 M Na-acetate, pH 5.2 and 2.5 volumes of ethanol. After incubation at −80 °C, samples were collected by centrifugation and suspended in double distilled water RNAse and DNAse free. The targeted deletion was identified by PCR using the Platinum Taq polymerase (10966018, Sigma) and the following primers: Tfr2-5LoxP-F: 5′-ggggtctacttcggagagtggtaag-3′; TfR2-5loxP-R: 5′-ctgagggttaggcaagaatggtgt-3′ and TfR2-3loxP-R 5′-ttctgccaacattctetccctctc-3′. The expected fragment sizes were: 201 bp for WT, 259 bp for TfR2-floxed and 294 bp for the deletion on the TfR2 allele.

### MPTP model

We used the sub-acute paradigm consisting of subcutaneous injection of 30 mg/kg free base MPTP (M0896, Sigma) dissolved in PBS or vehicle for 5 consecutive days in 12- and 52-week-old mice once a day [38]. During the procedure, however, we found that the 30 mg/kg regimen induced extreme mortality in the 52-week-old animals, and we therefore reduced the dose to 17.5 mg/kg. This dose was sufficient to elicit significant DA degeneration without mortality in the experiment. Animals were finally sacrificed 3 weeks after the last injection, when the degenerative process was already stabilized [80].

### In vitro synthesis of alpha-synuclein pre-formed fibrils (PFF)

alpha*-*Synuclein PFF have been generated from human alpha-synuclein monomer in assembly buffer (PBS, pH 7.0) as previously described [34, 37, 81]. Briefly, 4 mg of monomeric α-syn were shacked at 1000 rpm for 7 days at 37 °C. Fibrils were then aliquoted and stored at −80 °C in single use vials. The efficiency of the fibrillation reaction was verified with a Thioflavin T (25 µM, Sigma, T 3516) fluorimeter.

### Alpha-synuclein models: stereotaxic injection of AAV-alpha-synuclein and α-synuclein pre-formed fibrils (PFF)

Adenoviral-mediated overexpression of human alpha-synuclein was induced as previously described [33]. Briefly, recombinant adeno-associated virus (serotype 2 genome and serotype 6 capsid, AAV) was used to express human h-α-syn in the mouse substantia nigra. Gene expression was under the control of a human Synapsin 1 promoter and enhanced using a woodchuck hepatitis virus post-transcriptional regulatory element and a polyA signal downstream to the α-syn sequence. AAV-vector production, purification, concentration, and titration were performed by Sirion (Martinsried, Germany). AAV-synuclein or vehicle injections were performed at a rate of 0.4 µL/min using a Hamilton syringe fitted to a glass capillary, under isoflurane anesthesia. At the end of the procedure, the capillary was left in site for an additional 5 min before being retracted. Mice were injected with 1.5 µL of 8.0 × 10^12^ genome copies/mL AAV-α-syn into the right substantia nigra, with the following coordinates: 2.3 mm posterior and −1.1 mm lateral to Bregma, and 4.1 mm ventral to dura mater. Eight mice per group were used for this study.

PFF injections were performed into the striatum with the following coordinates: 0.2 mm anterior and 2.0 mm lateral to Bregma, and 2.6 mm ventral to the dura mater. 2.5 µL of sonicated PFF (5 µg in sterile PBS) were injected under isoflurane anesthesia according to standard procedures. Eight animals per group were used for this study.

### Immunohistochemistry

Free floating sections were first incubated in hydrogen peroxide (H_2_O_2_, Sigma-Aldrich, St. Louis, MO, USA) 3% in PBS for 30 min to block internal peroxidase activity, and subsequently in PBS-Triton X-100 0.2% (PBS-T) and normal horse serum (NHS) 10% for 1 h at RT. Specimens were then incubated for 24 h at 4 °C with mouse monoclonal anti-Tyrosine hydroxylase (1:4000, MAB318, Millipore, MA, USA) or with affinity purified anti human alpha-synuclein (1:3000, AB5038P, Millipore, Germany) in PBS-T and 1.5% NHS. After several washes with PBS-T, sections were incubated with biotinylated goat anti-rabbit IgG (1:500; BA 1000, Vector Laboratories, Burlingame, CA, USA), in PBS and 1% NHS for 1 h at RT. Immuno-complexes were revealed by Vectastain Elite ABC kit (PK 4000, Vector Laboratories, Burlingame, CA, USA), using 3,3′-diamino-benzidine (DAB Substrate kit for Peroxidase, SK 4100, Vector Laboratories, Burlingame, CA, USA) or by alkaline phosphatase (BCIP/NBT substrate alkaline phosphatase kit, SK5400, Vector Laboratories, Burlingame, CA, USA). Finally, sections were dehydrated and mounted with Eukitt (Kindler GmbH & Co). Images were acquired with the Nanozoomer 2.0 HT digital slide scanner and analyzed with the NDP viewer software (Hamamatsu).

### Quantification of striatal TH density

Striatal lesions in DAB-stained sections were calculated as the extension of the lesioned area, detected by the absence of TH staining, and expressed as percentage of TH immunopositive area measured on the contralateral side (for the AAV model) or on the PBS control mice (for the PFF model). A total of ten sections per animals were analyzed.

### Immunofluorescence

In immunofluorescence experiments, tissue sections were processed as described in the immunohistochemistry section with minor modifications. Floating sections were incubated overnight at 4 °C with primary antibodies. The following antibodies were used: mouse monoclonal anti-Tyrosine Hydroxylase (1:4000, MAB318, Millipore), sheep polyclonal anti-Tyrosine Hydroxylase (1:4000; NB300-110, Novus Biologicals), anti-phospho-α-Synuclein (S129; 1:1000; ab59264, Millipore, Germany), anti-MAC2 (1:1000; CL8942AP, Millipore), anti–GFAP (AB7260, 1:2000 Abcam).

Tissue sections were rinsed with PBS and then incubated for 2 h at RT in TBS containing 0.4% Triton X-100, 2% NHS, Alexa488 conjugated donkey anti-rabbit IgG 1:500 (Invitrogen), Alexa594 conjugated donkey anti-mouse IgG 1:500 (Invitrogen), CY3 conjugated anti-sheep IgG (1:500, Jackson). Image acquisition was performed with a Leica TCS SP5 confocal microscope.

### Unbiased stereological counts

Unbiased stereological estimation of the total number of DA cells in SNpc was performed using the optical fractionator method [82] and the STEREO INVESTIGATOR program on a Neurolucida computer-controlled microscopy system (Microbrightfield Inc., Williston, VT, USA). The edges of the SNc in the rostro-caudal axis were defined at all levels, with reference to a coronal atlas of the mouse brain [83]. TH-positive cells in the SNc of both hemispheres were counted in three sections, on comparable sections for all the subgroups of treatment throughout the entire nucleus. Counting frames (60 × 60 µm) were placed at the intersections of a grid (frame size 120 × 120 µm) that was randomly placed over the section. For counting, only those counting frames in which at least a part of the frame fell within the contour of the SNpc were used. TH-positive cell bodies were marked only if in focus. Guard volumes (3 µm from the top and 3 µm from the bottom of the section) were excluded from both surfaces to avoid the problem of lost caps. The reliability of the estimate was assessed by calculation of the coefficient of error as previously described [84].

### DAB enhanced Perls staining for ferric iron

To detect Fe^3+^ iron content, slices were washed two times with phosphate buffer (PB) 0.1 M and then incubated with equal volumes of HCl 20% and potassium ferrocynide (K_4_Fe(CN)_6_ × 3·H_2_O) 10% for 40 min to release Fe^3+^. After three washes in PB 0.1 M, sections were treated with NaN3 0.01 M and H_2_O_2_ 3% in PB 0.1 M for 15 min to inactivate endogenous peroxidases. After two washes with PB 0.1 M and two following with 0.05 M Tris-HCl, iron accumulation was revealed with 3,3′-diamino-benzidine (DAB Substrate kit for Peroxidase, SK 4100, Vector Laboratories, Burlingame, CA, USA) containing H_2_O_2_ 0.005% for 50 min. Slices were then washed two times with Tris-HCl 0.05 M and two times with PB 0.1 M before drying and being mounted with Eukitt (Kindler GmbH & Co.) or processed for immunohistochemistry as described above. Images were acquired with the Nanozoomer 2.0 HT digital slide scanner and analyzed with the NDP viewer software (Hamamatsu).

### Statistical analysis

Experiments were performed at least in three independent biological and at least two independent technical replicates. All analyses were performed using Graph Pad Prism version 7.03 for Windows (GraphPad Software, La Jolla, CA, USA). *P* values expressed as *P < 0.05; **P < 0.01, ***P < 0.001 were considered to be statistically significant; in the absence of indications, comparisons should be considered non-significant. Comparisons for two groups were calculated by unpaired two-tailed Student’s *t* tests and comparisons for more than two groups were calculated by either one- or two-way analysis of variance respectively followed by Dunnett’s and Bonferroni post-hoc test. All values are expressed as mean ± SEM.

## Supplementary information

Supplemental figure 1

Supplemental figure 2

Supplemental figure 3
