# Supplementary figures and images for: Gender biased neuroprotective effect of Transferrin Receptor 2 deletion in multiple models of Parkinson’s disease
Source: Cell Death Differ. 2020 Dec 16;28(5):1720–32. doi: 10.1038/s41418-020-00698-4 (PMC8166951; doi:10.1038/s41418-020-00698-4)

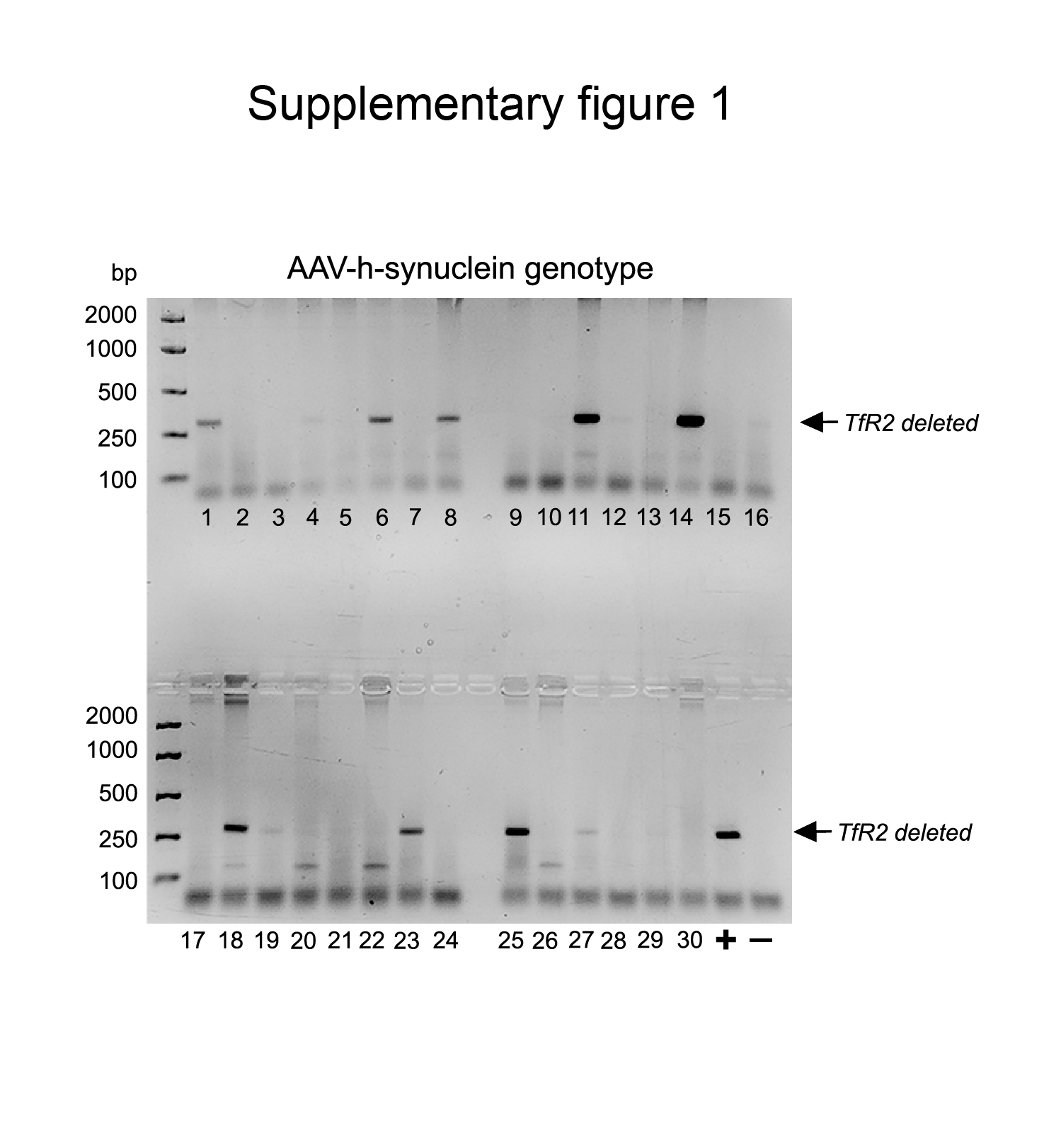

Supplement: Supplementary file 1 — Supplemental figure 1 [file 41418_2020_698_MOESM1_ESM.tif]

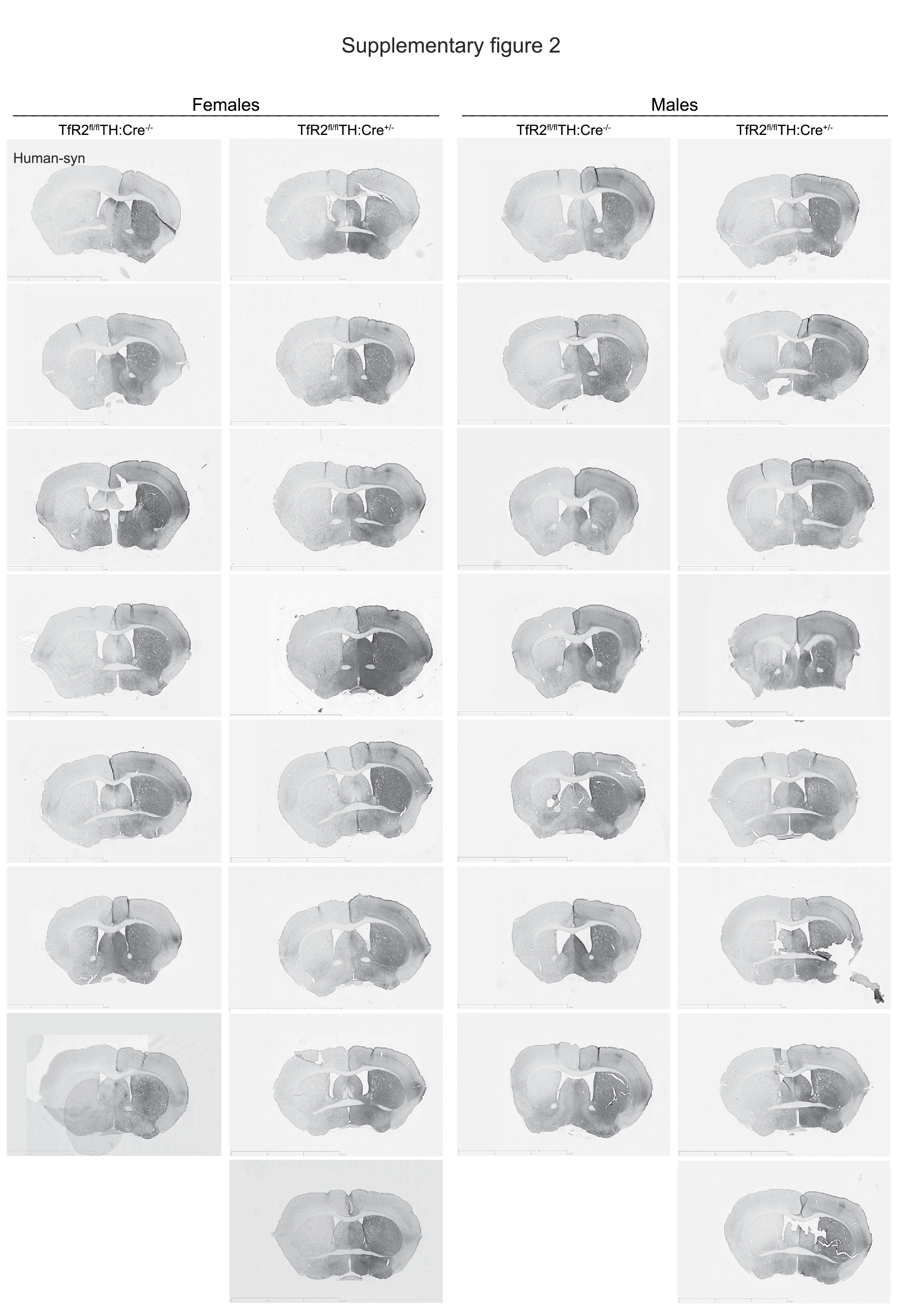

Supplement: Supplementary file 2 — Supplemental figure 2 [file 41418_2020_698_MOESM2_ESM.tif]

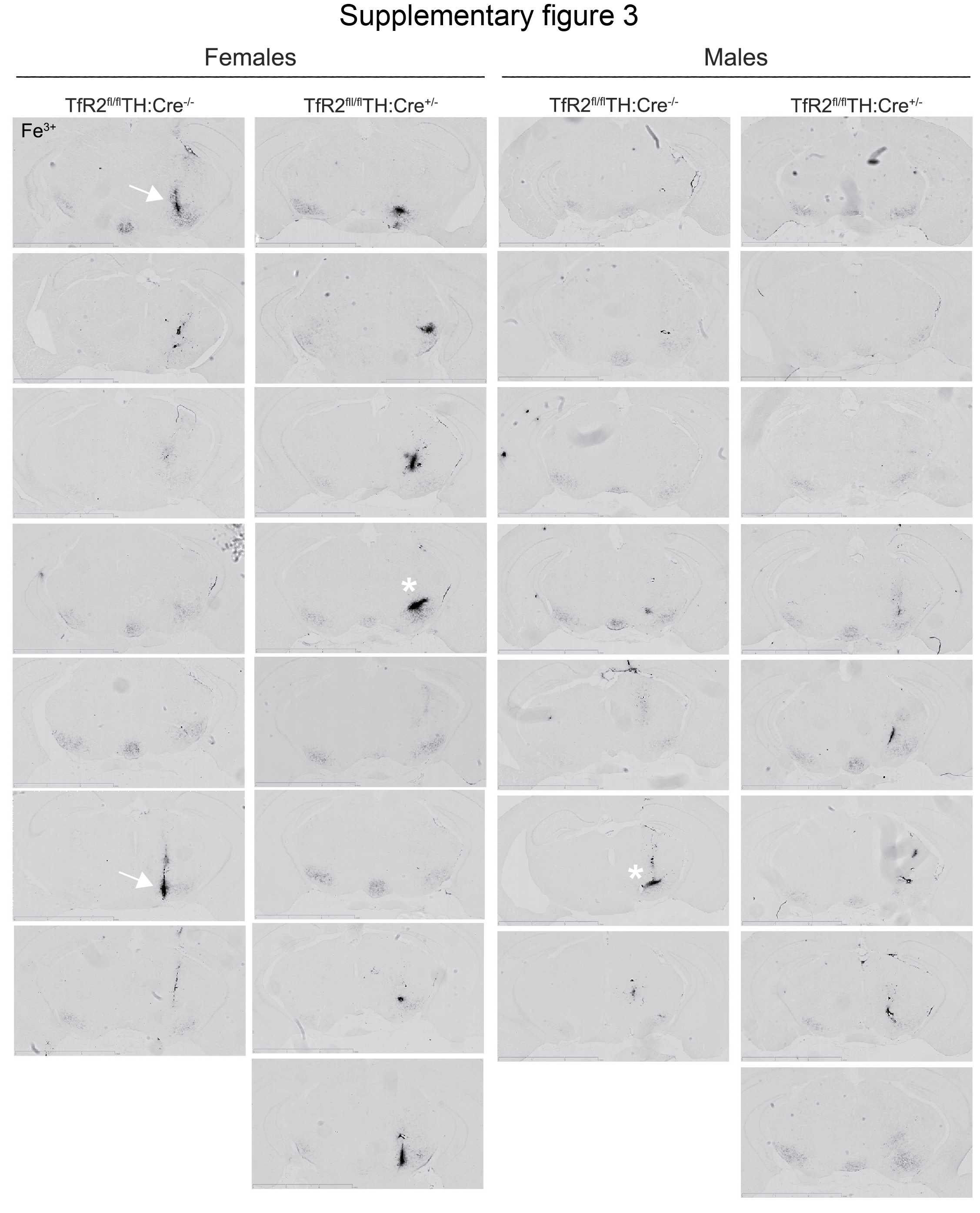

Supplement: Supplementary file 3 — Supplemental figure 3 [file 41418_2020_698_MOESM3_ESM.tif]
